# Irreversibility Analysis of Dissipative Fluid Flow Over A Curved Surface Stimulated by Variable Thermal Conductivity and Uniform Magnetic Field: Utilization of Generalized Differential Quadrature Method

**DOI:** 10.3390/e20120943

**Published:** 2018-12-07

**Authors:** Muhammad Idrees Afridi, Abderrahim Wakif, Muhammad Qasim, Abid Hussanan

**Affiliations:** 1 Department of Mathematics, COMSATS University Islamabad (CUI), Park Road, Tarlai Kalan, Islamabad 455000, Pakistan; 2 Laboratory of Mechanics, Faculty of Sciences Aïn Chock, Hassan II University, B.P. 5366 Mâarif, Casablanca 20000, Morocco; 3 Division of Computational Mathematics and Engineering, Institute for Computational Science, Ton Duc Thang University, Ho Chi Minh City 700000, Vietnam; 4 Faculty of Mathematics and Statistics, Ton Duc Thang University, Ho Chi Minh City 700000, Vietnam

**Keywords:** irreversibility analysis, generalized differential quadrature method (GDQM), heat transfer, variable thermal conductivity, energy and magnetic dissipation, curved surface

## Abstract

The effects of variable thermal conductivity on heat transfer and entropy generation in a flow over a curved surface are investigated in the present study. In addition, the effects of energy dissipation and Ohmic heating are also incorporated in the modelling of the energy equation. Appropriate transformations are used to develop the self-similar equations from the governing equations of momentum and energy. The resulting self-similar equations are then solved by the Generalized Differential Quadrature Method (GDQM). For the validation and precision of the developed numerical solution, the resulting equations are also solved numerically using the Runge-Kutta-Fehlberg method (RKFM). An excellent agreement is found between the numerical results of the two methods. To examine the impacts of emerging physical parameters on velocity, temperature distribution and entropy generation, the numerical results are plotted against the various values of physical flow parameters and discussed physically in detail.

## 1. Introduction

The first law of thermodynamics gives a quantitative estimate of heat and work interactions between some system and surroundings if the system undergoes a thermodynamic process or a cycle. However, it does not say whether the process or the cycle in a particular direction would occur or not. Further, the first law does not indicate whether conversion of energy from one form to another are performed perfectly or whether some forms are completely converted to others. The first law establishes the equivalence of heat and work and shows there is a fixed rate of exchange between heat and work. It does not talk about the conditions under which the transformations of energy are possible. Second law of thermodynamics puts a condition on the conversion of energy from one form to another. The second law states that it is not possible to convert heat energy completely into work. That part of heat energy which cannot be converted into work is known as unavailable energy and must be rejected as a low-grade energy. This means that, the availability of energy in a thermal system decreases. The phenomenon of increasing unavailable energy in a thermal process is called entropy generation. The thermal efficiency reduces with increasing entropy generation and therefore needs to examine the factors that reduces the entropy generation. Bejan [1] introduced the innovative idea of reducing the unavailable energy (entropy generation) in a convective heat transfer problem. After the pioneering work of Bejan [1], analysis of entropy generation in fluid flow problems are reported by many researchers. Recently, Afridi et al. [2] studied the entropy generation of carbon nanotubes CNTs nanofluids in a flow over a thin needle by incorporating the effects of nonlinear thermal radiation and viscous dissipation. Makinde [3] reported the effects of variable viscosity on inherent irreversibility in a flow over a flat plate with Newtonian heating and uniform magnetic field. The combined effects of linear thermal radiation and viscous dissipation on entropy generation in a Blasius flow are reported by Butt et al. [4]. Recently, Afridi and Qasim [5] examined the influences of frictional heating on entropy production rate in a three-dimensional flow. Entropy generation in a non-linear convection flow over a vertical plate with convective boundary condition and porous medium is studied by Vasu et al. [6]. Afridi et al. [7] studied the exact effects of viscous dissipation, Joule heating and heat transfer on entropy generation in a flow of Newtonian fluid over an elastic stretching boundary. The heat transfer and irreversibility analysis of nanofluid flow containing copper nanoparticles with water as a base fluid is reported by Butt et al. [8]. Makinde and Eegunjobi [9] studied the heat transfer effects in a couple stress fluid flow inside a vertical channel filled with porous medium with entropy analysis. Rashidi et al. [10] investigated the entropy generation in a nanofluid flow over a rotating disk under the influence of uniform magnetic field.

Boundary layer flows have significant number of applications in industrial and engineering processes such as extraction of polymer sheet, glass-fiber production, spinning of fibers, wire drawing, extruded plastic sheets, paper production, hot rolling and materials handling conveyors. Boundary layer flow is the corner stone of modern fluid dynamics due to vital application in manufacturing processes [11]. After the seminal work of Sakiadis [12], many researchers examined the boundary layer flow either by using analytic techniques or by utilizing various numerical methods. Crane [13] reported the exact solution of boundary layer flow driven by a stretching surface. Gupta and Gupta [14] reported the effects of transpiration on heat and mass transfer in a flow over a stretching boundary. Flow driven by a stretching cylinder is reported by Wang [15]. Wang [16] also reported the rotating fluid flow over a stretching boundary. The thin film flow over a stretching surface with variable fluid properties is studied by Dandapat et al. [17]. Vajravelu and Rollins [18] reported the flow of second grade fluid under the effect of magnetic field. Pal and Mondal [19] examined the effects of prescribed surface temperature (PST) and prescribed heat flux (PHF) on the mixed convection fluid with variable transport properties. The effects of energy dissipation with prescribed surface temperature on a power-law fluid flow driven by a permeable boundary are examined by Yazdi et al. [20]. Hsiao [21] analyzed the influences of viscous dissipation on viscoelastic fluid. The flow over a slender stretching sheet with Hall effects and variable fluid properties is studied by Vajravelu et al. [22]. Mixed convection flow of Casson fluid over a vertical flat surface is investigated by Vajravelu et al. [23]. Some of other recent studies on a boundary layer flow over a flat surface and curved surface are referenced in [24,25,26,27,28,29]. The effects of magnetic field on fluid flow are investigated in [30,31,32].

The objective of the present article is to study the heat transfer effects in a dissipative fluid flow over an elastic curved surface with variable thermal conductivity. One of the most important effect in the boundary layer flow knowns as viscous dissipation is also considered by adding the dissipation function the energy equation. Besides this, the entropy generation analysis is also performed. Numerical solutions of the transformed set of highly nonlinear differential equations are obtained and utilized to compute the entropy generation number. Generalized Differential Quadrature Method (GDQM) is used to get the numerical solutions. The numerical results are presented graphically and discussed.

## 2. Problem Formulation

**Figure 1:**
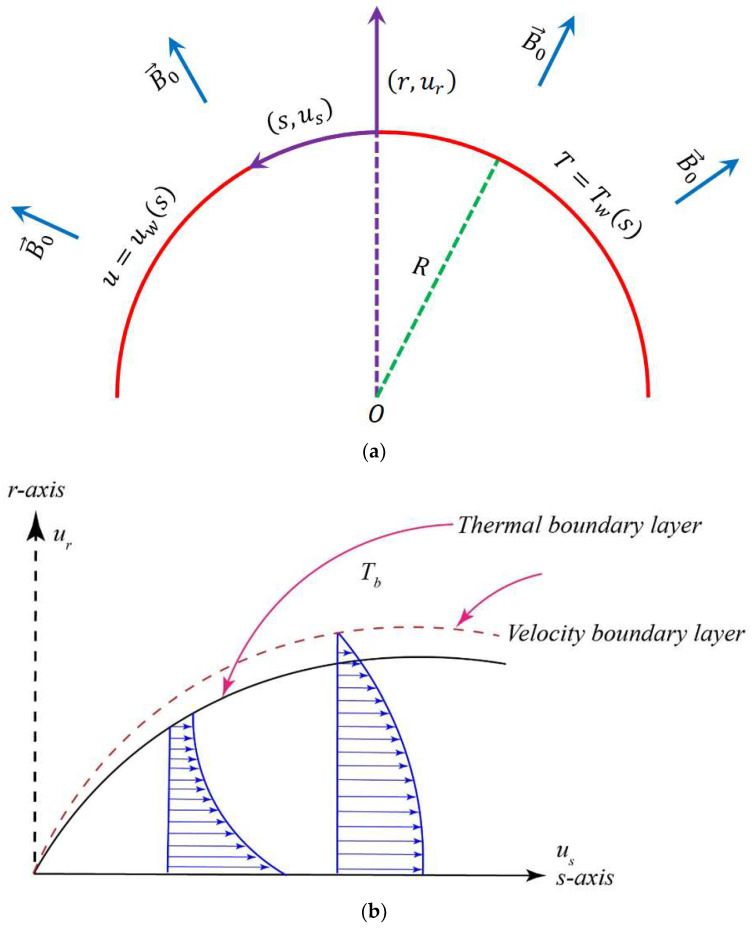
(**a**) Geometry of the flow domain. (**b**) Thermal and momentum boundary layer.

Let us consider a two-dimensional incompressible steady flow of a Newtonian electrically conducting fluid over a linearly curved stretching surface as schematically described in Figure 1. Moreover, this curved boundary (i.e., *r* = *R*) is taken to be coiled in a circle of radius *R*. In this investigation, we consider that the induced boundary layer flow is significantly affected by a radial magnetic field with constant strength *B*_0_, in which the fluid is flowing under the combined effects of viscous dissipation and Joule heating with the presence of a temperature dependent behavior for the thermal conductivity in the form k* = *k ω*(*T*). In order to define the flow geometry, we choose to employ the curvilinear coordinates (*r,s*) as the best way for modelling the present problem, where r is the radial coordinate measured from the center *O* of the curved surface, whereas s is the coordinate of the arc length along the flow direction. Furthermore, the stretching velocity and temperature of the curved sheet are taken to be in the form *u_w_*(*s*) = *u_o_s* and *T_w_*(*s*) = *T_b_* + *T_o_s*^2^, respectively, where *T_b_* represents the temperature of the bulk fluid, so that *u_o_* and *T_0_* are two dimensional constants.

Based on the Prandtl boundary layer approximations and the afore-mentioned assumptions, the basic equations of continuity, momentum and thermal energy are written as follows:

(1) $$\frac{\partial \;}{\partial r}\left({\bar{r}}_{}u_{r}\right)+R\frac{\partial u_{s}}{\partial s}=0\;,$$

(2) $$\frac{1}{\bar{r}}u_{s}^{2}=\frac{1}{\rho }\frac{\partial p}{\partial r}\;,$$

(3) $$\rho \left(u_{r}\frac{\partial u_{s}}{\partial r}+\frac{R}{\bar{r}}u_{s}\frac{\partial u_{s}}{\partial s}+\frac{1}{\bar{r}}u_{s}u_{r}\right)=-\frac{R}{\bar{r}}\frac{\partial p}{\partial s}+\mu \left(\frac{\partial^{2}u_{s}}{\partial r^{2}}+\frac{1}{\bar{r}}\frac{\partial u_{s}}{\partial r}-\frac{1}{{\bar{r}}^{2}}u_{s}\right)-\sigma B_{o}^{2}u_{s}\;,$$

(4) $$\rho c_{p}\left(u_{r}\frac{\partial T}{\partial r}+\frac{R}{\bar{r}}u_{s}\frac{\partial T}{\partial s}\right)=\frac{\;1\;}{r^{*}}\frac{\partial }{\partial r}\left({\bar{r}}_{}k^{*}\frac{\partial T}{\partial r}\right)+\mu {\left(\frac{\partial u_{s}}{\partial r}-\frac{1}{\bar{r}}u_{s}\right)}^{2}+\sigma B_{o}^{2}u_{s}^{2}.$$

Here, the symbols *ρ*, *μ, σ* and k* represents the thermo-physical properties of the electrically conducting fluid. These quantities denote the density, dynamic viscosity, electrical conductivity and thermal conductivity of the studied fluid, where $\bar{r}$ is the reduced radial variable, where $\bar{r}=r+R$.

Additionally, it is worth noting here that the characteristic function *ω*(*T*) mentioned above is taken in the form:

(5) $$\omega \left(T\right)=1+\tau \left(\frac{T-T_{b}}{T_{w}-T_{b}}\right),$$

where τ is an adjusted parameter showing the importance of the temperature dependence in the thermal conductivity k*.

For the present two-dimensional steady flow model, the associated physical boundary conditions are written in curvilinear coordinates as follows:

(6) $$u_{s}=u_{w},\;T=T_{w}\;\mathrm{at}\;r=0,$$

(7) $$u_{s}\to 0,\;\frac{\partial u_{s}}{\partial r}\to 0,\;T\to T_{b}\;\mathrm{as}\;r\to \infty .$$

Here, $u_{s}$ and $u_{r}$ are the velocity components in s and r directions, respectively, *B_o_* denotes the magnetic field strength, *p* and *T* show the pressure and temperature of the fluid, respectively, *T_w_* represents the temperature of the curved sheet, whereas *T_b_* indicates the temperature of the fluid in the stress free region.

Now, by introducing the following similarity transformations:

(8) $$\begin{array}{c} \chi ={\left(\frac{u_{o}\rho }{\mu \;}\right)}^{0.5}r,\;g^{\prime }\left(\chi \right)=\frac{u_{s}\left(r,s\right)}{u_{w}},\;g\left(\chi \right)=-{\left(\frac{\rho }{u_{o}\mu }\right)}^{0.5}\left(\frac{\bar{r}}{R}\right)u_{r}\left(r,s\right),\;\\ \theta =\frac{T-T_{b}}{T_{w}-T_{b}},\;P\left(\chi \right)=\frac{1}{\rho_{}u_{o}^{2}s^{2}}p, \end{array}$$

Equations (1)–(4) reduce to

(9) $$\frac{\partial P}{\partial \chi }=\frac{{g^{\prime }}^{2}}{\;h\;},$$

(10) $$\frac{2\kappa }{h}P=g^{\prime \prime \prime }+\frac{g^{\prime \prime }}{h}-\left(\frac{1}{h^{2}}+M\right)g^{\prime }+\frac{\;\kappa }{h}gg^{\prime \prime }+\frac{\kappa }{h^{2}}gg^{\prime }-\frac{\;\kappa }{h}{g^{\prime }}^{2},$$

(11) $$\frac{1}{\mathrm{Pr}}\left(1+\tau \theta \right)\left(\theta^{\prime \prime }+\frac{\theta^{\prime }}{h}\right)+\frac{\tau }{\mathrm{Pr}}{\theta^{\prime }}^{2}+\frac{\;\kappa }{h}\left(g\theta^{\prime }-2g^{\prime }\theta \right)+Ec{\left(g^{\prime \prime }-\frac{g^{\prime }}{h}\right)}^{2}+EcM{g^{\prime }}^{2}=0\;.$$

Here, κ denotes the curvature parameter, *Ec* and Pr represent the Eckert and Prandtl numbers, respectively, whereas *M* represents the magnetic parameter, where:

(12) $$\kappa =R{\left(\frac{u_{o}\rho }{\mu \;}\right)}^{0.5},\;Ec=\frac{u_{w}^{2}}{c_{p}\left(T_{w}-T_{b}\right)},\;\mathrm{Pr}=\frac{c_{p}\mu }{k},\;M=\frac{B_{o}^{2}\sigma }{u_{o}\rho }.$$

In this paper, we use the prime notation (e.g., *g’* or *θ’)* in Equations (8)–(11) as subscript to denote the derivative with respect to χ. Furthermore, the function *h* shown in Equations (9)–(11) is a linear function of the similarity variable χ, which is given by:

(13) h(χ)=χ+κ .

Also, after combining Equations (9) and (10), we get:

(14) $$g^{\prime \prime \prime \prime }+\frac{2}{h}g^{\prime \prime \prime }-{\bar{g}}_{1}{}_{}g^{\prime \prime }+{\bar{g}}_{2}g^{\prime }+\frac{\;\kappa }{h}\left(gg^{\prime \prime \prime }+\frac{g}{h}g^{\prime \prime }-\frac{1}{h}{g^{\prime }}^{2}-g^{\prime }g^{\prime \prime }-\frac{g}{h^{2}}g^{\prime }\right)=0,$$

where:

(15) $${\bar{g}}_{1}=\frac{1\;}{h^{2}}+M,$$

(16) $${\bar{g}}_{2}=\frac{1\;}{h^{3}}-\frac{M}{h}.$$

Upon making use of the transformations (8), the boundary conditions (6) and (7) become:

(17) $$g\left(0\right)=0,\;g^{\prime }\left(0\right)=1,\;\theta \left(0\right)=1,$$

(18) $$g^{\prime }\left(\chi \to \infty \right)\to 0,\;g^{\prime \prime }\left(\chi \to \infty \right)\to 0,\;\theta \left(\chi \to \infty \right)\to 0.$$

Furthermore, the important physical quantities of practical interest arising from this investigation are the local skin friction coefficient *Cf_s_* and the local Nusselt number *Nu_s_*, which are expressed by:

(19) Res0.5Cfs=g″(0)−1κg′(0),

(20) Res−0.5Nus=−θ′(0)−τθ′2(0).

Here, Re*_s_* represents the local Reynolds number, where Res=uos2/ν.

For more helpful simplifications, we can put:

(21) $$\left\{ \begin{array}{l} \chi =\chi_{\infty }{}_{}\eta ,\\ h\left(\chi \right)=h\left(\chi_{\infty }{}_{}\eta \right)=H\left(\eta \right),\\ {\bar{g}}_{1}\left(\chi \right)={\bar{g}}_{1}\left(\chi_{\infty }{}_{}\eta \right)={\bar{G}}_{1}\left(\eta \right),\\ {\bar{g}}_{2}\left(\chi \right)={\bar{g}}_{1}\left(\chi_{\infty }{}_{}\eta \right)={\bar{G}}_{2}\left(\eta \right),\\ g\left(\chi \right)=g\left(\chi_{\infty }{}_{}\eta \right)=G\left(\eta \right),\\ \theta \left(\chi \right)=\theta \left(\chi_{\infty }\eta \right)=\Theta \left(\eta \right). \end{array}\right.$$

Keeping in mind the above considerations, the derivatives of *g*(*χ*) and *θ*(*χ*) can be expressed as function of the derivatives of *G*(η) and Θ(*η*), respectively, as follows:

(22) $$\left\{ \begin{array}{l} g^{\left(n\right)}\left(\chi \right)=\frac{G^{\left(n\right)}\left(\eta \right)}{\chi_{{}_{\infty }}^{n}},\\ \theta^{\left(n\right)}\left(\chi \right)=\frac{\Theta^{\left(n\right)}\left(\eta \right)}{\chi_{{}_{\infty }}^{n}}, \end{array}\right.$$

where *n* denotes the integer-order derivative with respect to the spatial variables *χ* or *η*.

Accordingly, Equations (11) and (14) with the boundary conditions (17) and (18) can be written in the following general form:

(23) $$L_{G}\left(G\right)+N_{G}\left(G,\Theta \right)\;=0,\;$$

(24) $$L_{\Theta }\left(\Theta \right)+N_{\Theta }\left(G,\Theta \right)\;=0,\;$$

(25) $$G\left(\eta \right)=0,\;G^{\prime }\left(\eta \right)=\chi_{\infty },\;\Theta \left(\eta \right)=1,\;\mathrm{at}\;\eta =0,$$

(26) $$G^{\prime }\left(\eta \right)\to 0,\;G^{\prime \prime }\left(\eta \right)\to 0,\;\Theta \left(\eta \right)\to 0,\;\mathrm{as}\;\eta \to 1,$$

in which:

(27) $$L_{G}\left(G\right)=\;G^{\prime \prime \prime \prime }+\frac{2\chi_{\infty }}{H}G^{\prime \prime \prime }-\chi_{\infty }^{2}{\bar{G}}_{1}G^{\prime \prime }+\chi_{\infty }^{3}{\bar{G}}_{2}G^{\prime },$$

(28) $$L_{\Theta }\left(\Theta \right)=\frac{\chi_{\infty }^{2}}{\mathrm{Pr}}\Theta^{\prime \prime }+\frac{\chi_{\infty }^{3}}{\mathrm{Pr}H}\Theta^{\prime },$$

(29) $$N_{G}\left(G,\Theta \right)=\frac{\chi_{\infty }\kappa }{H}\left(GG^{\prime \prime \prime }+\frac{\chi_{\infty }G}{H}G^{\prime \prime }-\frac{\chi_{\infty }}{H}{G^{\prime }}^{2}-G^{\prime }G^{\prime \prime }-\frac{\chi_{\infty }^{2}G}{H^{2}}G^{\prime }\right),$$

(30) $$N_{\Theta }\left(G,\Theta \right)=\left\{\begin{array}{l} \frac{\chi_{\infty }^{2}\tau }{\mathrm{Pr}}{\Theta^{\prime }}^{2}+\frac{\chi_{\infty }^{3}\tau_{}\Theta }{\mathrm{Pr}H}\Theta^{\prime }+\frac{\chi_{\infty }^{2}\tau }{\mathrm{Pr}}\Theta \Theta^{\prime \prime }+\frac{\chi_{\infty }^{3}\kappa_{}G}{H}\Theta^{\prime }-\\ \frac{2\chi_{\infty }^{3}\kappa_{}\Theta }{H}G^{\prime }+Ec_{}{G^{\prime \prime }}^{2}-\frac{2\chi_{\infty }Ec}{H}G^{\prime }G^{\prime \prime }+\chi_{\infty }^{2}Ec{\bar{G}}_{1}{G^{\prime }}^{2} \end{array}\right\}.$$

Here, $\chi_{\infty }^{}$ represents the optimum value of the boundary layer thickness, which ensures our numerical findings are approached asymptotically to their exact values.

After substituting Equation (21) into Equations (19) and (20), the physical quantities (Re*_s_*)^0.5^ *Cf_s_* and (Re*_s_*)^−0.5^*Nu_s_* become:

(31) Res0.5Cfs=1 χ∞2G″(0)−1χ∞κG′(0),

(32) Res−0.5Nus=−1χ∞Θ′(0)−τχ∞2Θ′2(0).

## 3. Analysis of Entropy Production

As is well known, the local volumetric rate of entropy generation $\xi_{g}$ of a fluidic system in the presence of viscous dissipation and Ohmic heating is given by:

(33) $$\xi_{g}=\xi_{t}+\xi_{f}+\xi_{m},$$

where $\xi_{t}$ represents the entropy generation due to heat transfer across a finite temperature difference, $\xi_{f}$ shows the local entropy generation due to viscous dissipation and $\xi_{m}$ characterizes the local entropy generation due to the presence of Lorentz force, where:

(34) $$\xi_{t}=\frac{k\omega \left(T\right)}{T^{2}}{\left(\frac{\partial T}{\partial r}\right)}^{2},$$

(35) $$\xi_{f}=\frac{\mu }{T}{\left(\frac{\partial u_{s}}{\partial r}-\frac{u_{s}}{r^{*}}\right)}^{2},$$

(36) $$\xi_{m}=\frac{\sigma B_{o}^{2}}{T}u_{s}^{2}.$$

By considering the following characteristic entropy generation:

(37) $$\xi_{c}=\frac{k_{}u_{o}}{\nu },$$

the entropy generation in dimensionless form can be written as follows:

(38) $$N_{s}=\frac{\xi_{g}}{\xi_{c}}=\underset{Thermal\;contribution}{\underset{︸}{\frac{\left(1+\tau \theta \right)}{{\left(\theta +\lambda \right)}^{2}}{\theta^{\prime }}^{2}}}+\underset{Frictional\;contribution}{\underset{︸}{\frac{Ec\mathrm{Pr}}{\left(\theta +\lambda \right)}{\left(g^{\prime \prime }-\frac{g^{\prime }}{h\left(\xi \right)}\right)}^{2}}}+\underset{Magnetic\;contribution}{\underset{︸}{\frac{MEc\mathrm{Pr}}{\left(\theta +\lambda \right)}{g^{\prime }}^{2}}},$$

where $\lambda =T_{b}/\left(T_{w}-T_{b}\right)$ denotes the temperature difference parameter.

By virtue of Equation (21), the entropy generation *N_s_* reduces to:

(39) $$N_{s}=\frac{\left(1+\tau_{}\Theta \right)}{\chi_{\infty }^{2}{\left(\Theta +\lambda \right)}^{2}}{\Theta^{\prime }}^{2}+\frac{Ec\mathrm{Pr}}{\chi_{\infty }^{4}\left(\Theta +\lambda \right)}{\left(G^{\prime \prime }-\chi_{\infty }\frac{G^{\prime }}{H\;}\right)}^{2}+\frac{MEc\mathrm{Pr}}{\chi_{\infty }^{2}\left(\Theta +\lambda \right)}{G^{\prime }}^{2}.$$

## 4. Solution Methodology

The boundary layer flow model induced over the curved stretching surface *r* = *R* can be regarded as a complicated two-point boundary value problem. As mentioned previously in Equations (23) and (24), the present physical problem is governed by a set of ordinary differential equations ODEs, which are highly nonlinear. From the mathematical point of view, the flow and heat transfer characteristics of the studied fluid are extremely difficult to be found analytically as closed form solutions. Therefore, for solving this kind of physical problems, it is more recommended here to adopt a powerful numerical method in terms of accuracy and efficiency to predict approximate numerical solutions for Equations (23) and (24) along with the boundary conditions (25) and (26). Hence, in order to achieve this objective and ensure enough accuracy, the resulting ODEs are handled numerically by discretizing the present boundary layer equations using generalized differential quadrature method (GDQM) with the following non-uniform grid points:

(40) $$\eta_{i}=\frac{1}{2}-\frac{1}{2}cos\left(\frac{\pi i-\pi }{N-1}\right)\;.$$

Here, 1≤i≤N and *η*_1_ ≤ *η_1_* ≤ *η_N_*, where *η*_1_ = 0 and *η_N_ = 1*.

Accordingly, the discretized form of the derivatives of the functions *G*(*η*) and Θ(*η*) with respect to the variable *η* at a collocation point $\eta_{i}$ are defined as follows:

(41) $$\left\{ \begin{array}{l} G^{\left(n\right)}\left(\eta_{i}\right)=\sum_{j=1}^{N}d_{ij}^{\left(n\right)}G\left(\eta_{j}\right)=\sum_{j=1}^{N}d_{ij}^{\left(n\right)}G_{j}\;,\\ \Theta^{\left(n\right)}\left(\eta_{i}\right)=\;\sum_{j=1}^{N}d_{ij}^{\left(n\right)}\Theta \left(\eta_{j}\right)=\sum_{j=1}^{N}d_{ij}^{\left(n\right)}\Theta_{j}, \end{array}\right.$$

Here, $d_{ij}^{\left(n\right)}$ are the weighting coefficients for the *n^th^*-order derivative and *N* is the total number of collocation points, where *i* and *j* are integers varying from 1 to *N.*

In order to provide enough information about the proposed numerical method, the interested readers can refer to [33,34] and the reference therein. According to the pioneer work of Shu [33], the weighting coefficients $d_{ij}^{\left(1\right)}$ for the first-order derivative discretization can be expressed as follows:

(42) $$\{\begin{array}{ll} d_{ij}^{\left(1\right)}=\frac{\overset{N}{\underset{k=1,\;k\neq i}{\prod }}\left(\eta_{i}-\eta_{k}\right)}{\left(\eta_{i}-\eta_{j}\right)\overset{N}{\underset{k=1,\;k\neq j}{\prod }}\left(\eta_{j}-\eta_{k}\right)}, & \mathrm{for}\;i\neq j\;,\;\\ d_{ij}^{\left(1\right)}=-\overset{N}{\underset{j=1,j\neq i}{\sum }}d_{ij}^{\left(1\right)}, & \mathrm{for}\;i=j\;,\; \end{array}$$

where 1 ≤ *i, j ≤ N*.

Similarly, the weighting coefficients $d_{ij}^{\left(n\right)}$ for the higher-order derivatives can be found numerically using the following recurrence relations:

(43) $$\{\begin{array}{ll} d_{ij}^{\left(n\right)}=n\left(d_{ii}^{\left(n-1\right)}d_{ij}^{\left(1\right)}-\frac{d_{ij}^{\left(n-1\right)}}{\eta_{i}-\eta_{j}}\right), & \mathrm{for}\;i\neq j\;,\\ d_{ij}^{\left(n\right)}=-\overset{N}{\underset{j=1,j\neq i}{\sum }}d_{ij}^{\left(n\right)}, & \mathrm{for}\;i=j\;,\; \end{array}$$

where 1 ≤ *I, j ≤ N* and *n* ≥ 2.

Therefore, after discretization of Equations (23)–(26), the functions H(η), ${\bar{G}}_{1}\left(\eta \right)$, ${\bar{G}}_{2}\left(\eta \right)$, G(η) and Θ(η) are approximated in each collocation point $\eta_{i}$ by $H_{i}$, ${\bar{G}}_{1i}$, ${\bar{G}}_{2i}$, $G_{i}$ and $\Theta_{i}$, respectively. Consequently, the discretized form of our problem is given as follows:

(44) $$(S)\{\begin{array}{l} G_{1}=0,\;\\ \sum_{j=1}^{N}d_{1j}^{\left(1\right)}G_{j}-\chi_{\infty }=0,\\ L_{G_{i}}\left(G_{i}\right)+N_{G_{i}}\left(G_{i},\Theta_{i}\right)\;=0\;,\;\mathrm{for}\;3\leq i\leq N-2,\\ \sum_{j=1}^{N}d_{Nj}^{\left(1\right)}G_{j}=0,\\ \sum_{j=1}^{N}d_{Nj}^{\left(2\right)}G_{j}=0,\\ \Theta_{1}-1=0.\\ L_{\Theta_{i}}\left(\Theta_{i}\right)+N_{\Theta_{i}}\left(G_{i},\Theta_{i}\right)\;=0\;,\;\mathrm{for}\;2\leq i\leq N-1\\ \Theta_{N}=0,\; \end{array}$$

in which:

(45) $$L_{G_{i}}\left(G_{i}\right)=\left\{\begin{array}{l} \sum_{j=1}^{N}d_{ij}^{\left(4\right)}G_{j}+\frac{2\chi_{\infty }}{H_{i}}\left(\sum_{j=1}^{N}d_{ij}^{\left(3\right)}G_{j}\right)-\\ \chi_{\infty }^{2}{\bar{G}}_{1i}\left(\sum_{j=1}^{N}d_{ij}^{\left(2\right)}G_{j}\right)+\chi_{\infty }^{3}{\bar{G}}_{2i}\left(\sum_{j=1}^{N}d_{ij}^{\left(1\right)}G_{j}\;\right) \end{array}\right\}\;,$$

(46) $$L_{\Theta_{i}}\left(\Theta_{i}\right)=\frac{\chi_{\infty }^{2}}{\mathrm{Pr}}\left(\sum_{j=1}^{N}d_{ij}^{\left(2\right)}\Theta_{j}\right)+\frac{\chi_{\infty }^{3}}{\mathrm{Pr}H_{i}}\left(\sum_{j=1}^{N}d_{ij}^{\left(1\right)}\Theta_{j}\right),$$

(47) $$N_{G_{i}}\left(G_{i},\Theta_{i}\right)=\frac{\chi_{\infty }\kappa }{H_{i}}\{\begin{array}{l} G_{i}\left(\sum_{j=1}^{N}d_{ij}^{\left(3\right)}G_{j}\right)+\frac{\chi_{\infty }G_{i}}{H_{i}}\left(\sum_{j=1}^{N}d_{ij}^{\left(2\right)}G_{j}\right)-\frac{\chi_{\infty }}{H_{i}}\left(\sum_{j=1}^{N}d_{ij}^{\left(1\right)}G_{j}\right)\left(\sum_{j=1}^{N}d_{ij}^{\left(1\right)}G_{j}\right)-\\ \left(\sum_{j=1}^{N}d_{ij}^{\left(1\right)}G_{j}\right)\left(\sum_{j=1}^{N}d_{ij}^{\left(2\right)}G_{j}\right)-\frac{\chi_{\infty }^{2}G_{i}}{H_{i}^{2}}\left(\sum_{j=1}^{N}d_{ij}^{\left(1\right)}G_{j}\right)\; \end{array}\}$$

(48) $$N_{\Theta_{i}}\left(G_{i},\Theta_{i}\right)=\{\begin{array}{l} \frac{\chi_{\infty }^{2}\tau }{\mathrm{Pr}}\left(\sum_{j=1}^{N}d_{ij}^{\left(1\right)}\Theta_{j}\right)\left(\sum_{j=1}^{N}d_{ij}^{\left(1\right)}\Theta_{j}\right)+\frac{\chi_{\infty }^{3}\tau_{}\Theta_{i}}{\mathrm{Pr}H_{i}}\left(\sum_{j=1}^{N}d_{ij}^{\left(1\right)}\Theta_{j}\right)+\;\\ \frac{\chi_{\infty }^{2}\tau }{\mathrm{Pr}}\Theta_{i}\left(\sum_{j=1}^{N}d_{ij}^{\left(2\right)}\Theta_{j}\right)+\frac{\chi_{\infty }^{3}\kappa_{}G_{i}}{H_{i}}\left(\sum_{j=1}^{N}d_{ij}^{\left(1\right)}\Theta_{j}\right)-\frac{2\chi_{\infty }^{3}\kappa_{}\Theta_{i}}{H_{i}}\left(\sum_{j=1}^{N}d_{ij}^{\left(1\right)}G_{j}\right)+\\ Ec\left(\sum_{j=1}^{N}d_{ij}^{\left(2\right)}G_{j}\right)\left(\sum_{j=1}^{N}d_{ij}^{\left(2\right)}G_{j}\right)-\frac{2\chi_{\infty }Ec}{H_{i}}\left(\sum_{j=1}^{N}d_{ij}^{\left(1\right)}G_{j}\right)\left(\sum_{j=1}^{N}d_{ij}^{\left(2\right)}G_{j}\right)+\\ \chi_{\infty }^{2}Ec{\bar{G}}_{1i}\left(\sum_{j=1}^{N}d_{ij}^{\left(1\right)}G_{j}\right)\left(\sum_{j=1}^{N}d_{ij}^{\left(1\right)}G_{j}\right) \end{array}\}$$

Here:

(49) $$H_{i}=\left(\kappa +\frac{\chi_{\infty }}{2}\right)\;-\frac{\chi_{\infty }}{2}cos\left(\frac{\pi i-\pi }{N-1}\right).$$

(50) $${\bar{G}}_{1i}=\frac{1\;}{H_{i}^{2}}+M,$$

(51) $${\bar{G}}_{2i}=\frac{1\;}{H_{i}^{3}}-\frac{M}{H_{i}}.$$

As shown in Equation (44), the algebraic nonlinear system (*S*) is constituted by 2N equations. This nonlinear system can be solved accurately by means of Newton-Raphson iterative scheme (NRIS). Hence, thanks to this technique, the dimensionless quantities (Re*_s_*)^0.5^ *Cf_s_* and (Re*_s_*)^−0.5^*Nu_s_* shown in Equations (31) and (32) can be computed numerically using the following expressions:

(52) Res0.5Cfs=1χ∞2(∑j=1Nd1j(2)Gj)−1χ∞κ(∑j=1Nd1j(1)Gj),

(53) Res−0.5Nus=−1χ∞(∑j=1Nd1j(1)Θj)−τχ∞2(∑j=1Nd1j(1)Θj)(∑j=1Nd1j(1)Θj).

From the methodological point of view, we take $\chi_{\infty }=10$ and *N =* 70 as the best selected values during all subsequent analyses, in order to find out significant numerical results with an absolute accuracy of the order of $10^{-8}$. Moreover, the average CPU time taken to compute the skin friction coefficient Re*_s_*^0.5^*Cf_s_* and the rate of heat transfer Re*_s_*^−0.5^*Nu_s_* by GDQM is generally no more than 10 s.

## 5. Results and Discussion

The transformed set of differential equations that govern the flow are highly nonlinear and thereby solved numerical by applying generalized differential quadrature method (GDQM) to quantify the influences of different physical flow parameter. The impacts of dimensionless flow parameters such as magnetic parameter *M*, curvature parameter κ, Eckert number *Ec*, Prandtl number Pr, variable thermal conductivity parameter τ and temperature difference parameter λ on velocity *g’*(*χ*), temperature *θ*(*χ*) and entropy generation *N_s_* are depicted in different graphs. In order to verify the accuracy of our numerical scheme, the local skin friction coefficient and Nusselt number are also computed using Runge-Kutta Fehlberg method (RKFM) as shown in Table 1. The comparison shows an excellent agreement and hence validates our numerical simulation. Table 1 also illustrates the influences of physical flow parameters on local skin friction coefficients and Nusselt number. It is inferred from Table 1 that skin friction coefficient increases with rising values of magnetic parameter and decreases with curvature parameter. The Eckert number, Prandtl number and variable thermal conductivity have no influence on skin friction coefficient. We also observed that the local Nusselt number decreases with rising values of magnetic parameter, Eckert number and variable thermal conductivity parameter. Further, it is noted that, local Nusselt number enhances with increasing values of curvature parameter and Prandtl number.

**Figure 2:**
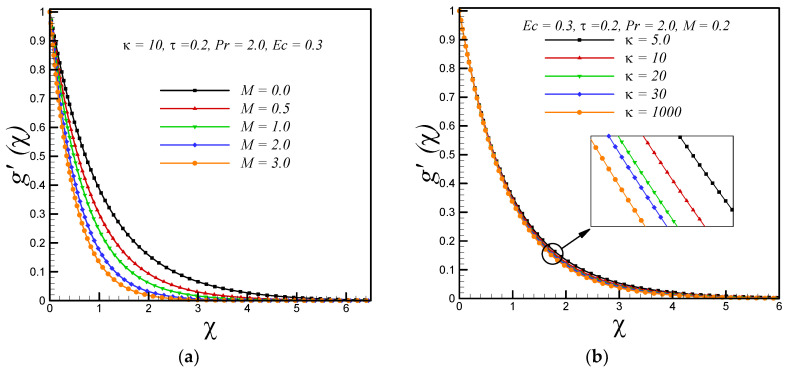
Impacts on velocity profile *g’*(*χ*) with variations in (**a**) magnetic parameter *M* and (**b**) curvature parameter κ.

**Figure 3:**
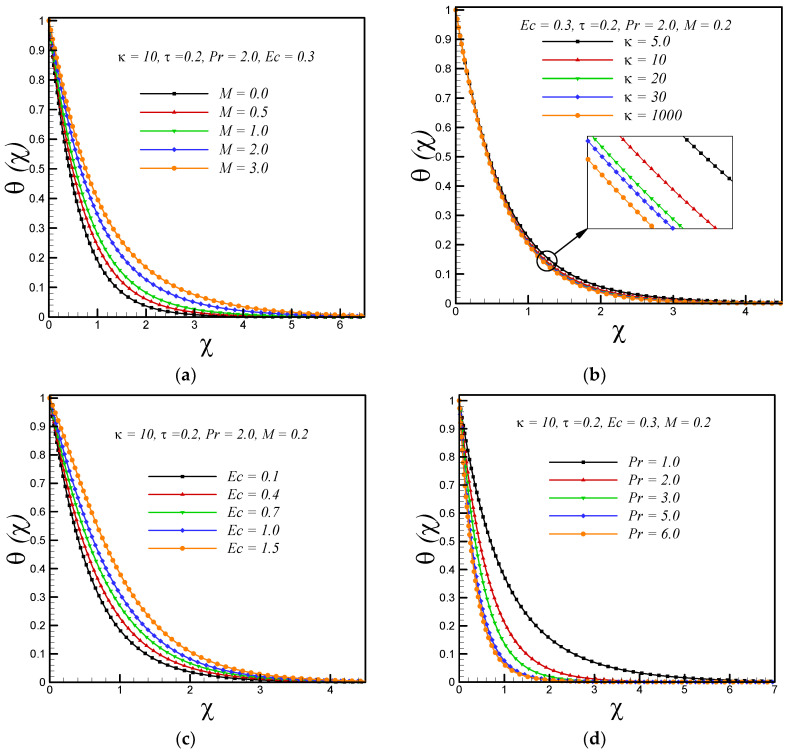

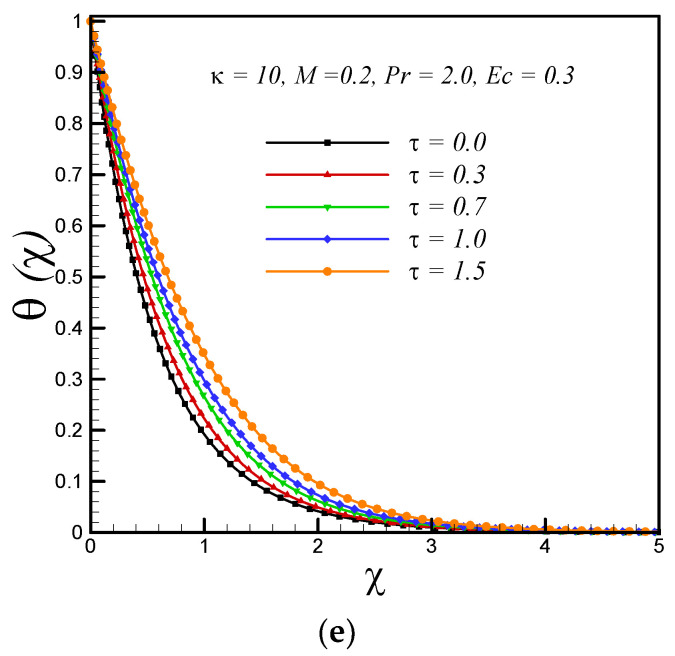
Impacts on temperature profile *θ*(*χ*)with variations in (**a**) magnetic parameter *M* (**b**) curvature parameter κ (**c**) Eckert number *Ec* (**d**) Prandtl number Pr and (**e**) variable thermal conductivity parameter τ.

**Figure 4:**
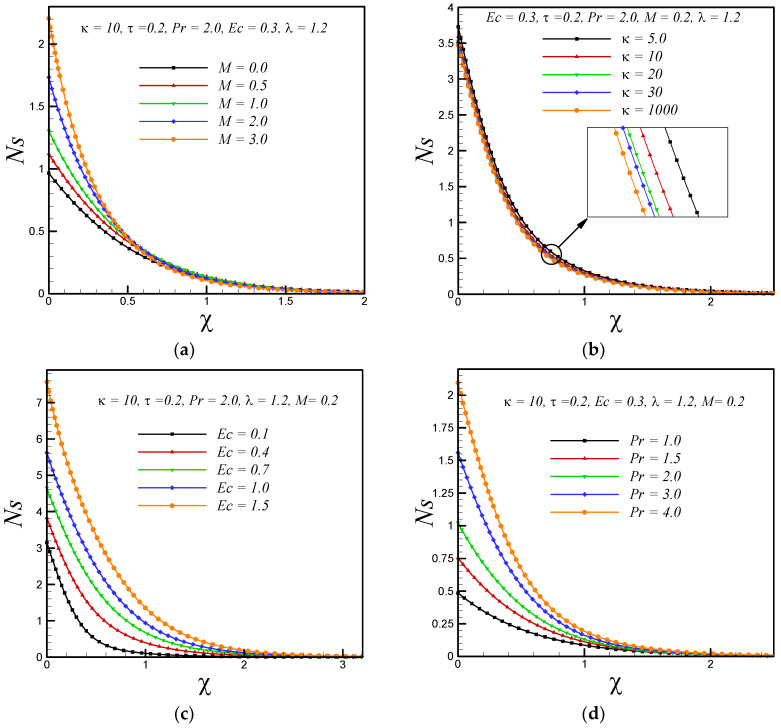

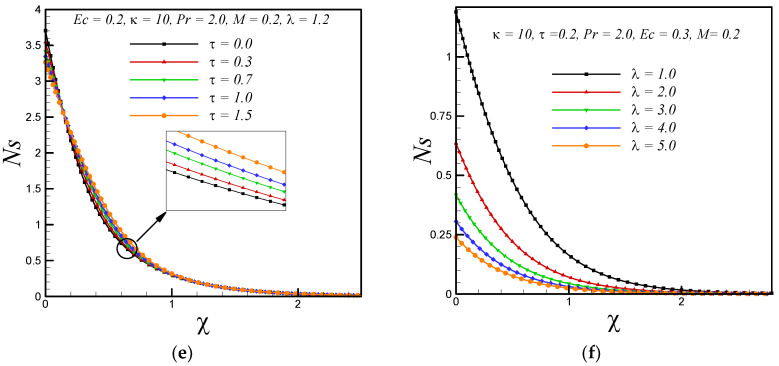
Impacts on entropy generation number *Ns* with variations in (**a**) magnetic parameter *M* (**b**) curvature parameter κ (**c**) Eckert number *E_C_* (**d**) Prandtl number Pr (**e**) variable thermal conductivity parameter τ and (**f**) temperature difference parameter *λ*.

Figure 2a,b represent the effects of magnetic and curvature parameter on velocity profile *g’*(*χ*) respectively. It is observed that motion of fluid decelerates with increasing strength of applied magnetic field. This is because a resistive force knows as Lorentz force enhances with increasing strength of applied magnetic field. It is also observed that velocity decreases with rising values of curvature parameter κ. Further, it is found that for fixed value of χ the thickness of boundary layer is thick for flow over a curved boundary as compared to flat surface (κ→∞). The effects of magnetic parameter(*M*), curvature parameter (*κ*), Eckert number (Ec), Prandtl number (Pr) and variable thermal conductivity parameter (*τ*) on temperature profile *θ*(*χ*) are shown in Figure 3a–e respectively. It is inferred from Figure 3a,b that temperature rises with increasing values of magnetic and curvature parameter respectively. This is because, the phenomenon of Ohmic heating increases with rising values of M and thus leads to rise the fluid temperature. We also observed that for fixed value of similarity variable *χ* the thickness of thermal boundary layer is thin for the fluid flow past over a flat stretching surface (κ→∞) as compared to flow over a curved stretching surface. The Eckert number *Ec* is measure of the frictional forces between the fluid layers, therefore, with increasing Eckert number the frictional heating enhances and leads to rise the fluid temperature as presented in Figure 3c. The decreasing behavior of temperature with increasing values of Prandtl number is due to the fact that thermal diffusivity decreases with increasing Prandtl number and consequently leads to drop the fluid temperature as shown in Figure 3d. The thermal conductivity of fluid increase with rising values of variable thermal conductivity parameter (*τ*), therefore, the temperature of fluid rises with increasing *τ* as shown in Figure 3e. Figure 4a shows that entropy generation enhances with enhancing the strength of applied magnetic field. This is due to the dissipative nature of the Lorentz force. In addition, it is noticed that the rate of entropy generation is maximum at the curved boundary. Figure 4b illustrates that entropy generation reduce with increasing values of curvature parameter κ. Further, no significant effects are observed at the surface of curved boundary. In addition, the rate of entropy generation is less in the flow over a flat boundary (κ→∞) as compared to the curved one. Figure 4c,d demonstrate the variations of entropy generation *N_S_* with Eckert number *Ec* and Prandtl number Pr, respectively. We found that entropy enhances with rising values of *Ec* and Pr. Significant effects are observed at the curved boundary and this is due to the presence of high thermal gradients at the surface of curved boundary. Figure 4e displays that, as the variable thermal conductivity parameter τ rises, the entropy generation *N_S_* enhance slightly at the surface of curved boundary and its vicinity. Furthermore, it is found that entropy generation *N_S_* decreases after certain vertical distance from the surface of stretching curved surface. Figure 4f demonstrates that, as temperature difference parameter *λ* increases, entropy generation *N_S_* reduces, therefore, to minimize the entropy generation inside the boundary layer, it is suggested to reduce the operating temperature ΔT (increase *λ*).

## 6. Closing Remarks

In the present investigation, we utilized the generalized differential quadrature method (GDQM) to get the numerical solutions of the reduced set of governing nonlinear differential equations. The impacts of different physical flow parameters are investigated by plotting various graphs. Following are the key outcomes of the present study.

- The local skin friction coefficient enhances with magnetic parameter and reduces with increasing curvature parameter.
- With an increase in magnetic parameter, Eckert number and variable thermal conductivity parameter, the local Nusselt number reduces but it enhances with rising values of curvature parameter and Prandtl number.
- The fluid motion decelerates with increasing *M* and curvature parameter κ.
- With rising values of magnetic parameter, Eckert number and variable thermal conductivity parameter, the temperature of fluid rises whereas decrement in temperature is observed with increasing values of Prandtl number and curvature parameter.
- Less entropy is generated in the flow past over a flat stretching boundary as compared to the flow over a curved surface.
- By increasing the curvature and temperature difference parameter, the entropy generation Ns reduces.
- With enhancing the values of magnetic parameter, Eckert number, Prandtl number and variable thermal conductivity parameter, *Ns* increases.

## Figures and Tables

**Table 1:** Present numerical results for the skin friction coefficient Re*_s_*^0.5^*Cf_s_* and the rate of heat transfer Re*_s_*^−0.5^*Nu_s_* at the curved surface by GDQM and RKFM, for various values of the physical parameters *M*, *κ*, *Ec,* Pr and *τ*.

*M*	*κ*	*Ec*	Pr	*τ*	*GDQM		*RKFM	
					−Res0.5Cfs	Res−0.5Nus	−Res0.5Cfs	Res−0.5Nus
0.0	10	0.3	2.0	0.2	1.0734886	1.0956346	1.0734886	1.0956346
0.5					1.3279849	1.0182902	1.3279849	1.0182902
1.0					1.5302913	0.9433763	1.5302913	0.9433763
2.0					1.8601286	0.7956016	1.8601286	0.7956016
3.0					2.1338460	0.6495366	2.1338460	0.6495366
0.2	5	0.3	2.0	0.2	1.2856525	1.0580225	1.2856526	1.0580225
	10				1.1846573	1.0641428	1.1846573	1.0641428
	20				1.1386292	1.0659353	1.1386292	1.0659353
	30				1.1239341	1.0663482	1.1239341	1.0663482
	1000				1.0963201	1.0668915	1.0963201	1.0668915
0.2	10	0.1	2.0	0.2	1.1846573	1.1176921	1.1846573	1.1176921
		0.4			1.1846573	1.0339380	1.1846573	1.0339380
		0.7			1.1846573	0.9295582	1.1846573	0.9295582
		1.0			1.1846573	0.8044534	1.1846573	0.8044534
		1.5			1.1846573	0.5496236	1.1846573	0.5496236
0.2	10	0.3	1.0	0.2	1.1846573	0.8221439	1.1846573	0.8221439
			2.0		1.1846573	1.0641428	1.1846573	1.0641428
			3.0		1.1846573	1.1801381	1.1846573	1.1801381
			5.0		1.1846573	1.2499780	1.1846573	1.2499781
			6.0		1.1846573	1.2391867	1.1846573	1.2391866
0.2	10	0.3	2.0	0.0	1.1846573	1.7356948	1.1846573	1.7356948
				0.3	1.1846573	0.8201182	1.1846573	0.8201182
				0.7	1.1846573	0.1759590	1.1846573	0.1759590
				1.0	1.1846573	−0.1120986	1.1846573	−0.1120984
				1.5	1.1846573	−0.4142181	1.1846573	−0.4142179

*GDQM: Generalized differential quadrature method; *RKFM: Runge-Kutta-Fehlberg method

## References

[1] Bejan A. (1999). The method of entropy generation minimization. Energy and the Environment.

[2] Afridi M.I., Tlili I., Qasim M., Khan I. (2018). Nonlinear Rosseland thermal radiation and energy dissipation effects on entropy generation in CNTs suspended nanofluids flow over a thin needle. Bound. Value Probl..

[3] Makinde O.D. (2011). Second law analysis for variable viscosity hydromagnetic boundary layer flow with thermal radiation and Newtonian heating. Entropy.

[4] Butt A.S., Munawar S., Ali A., Mehmood A. (2012). Entropy generation in the Blasius flow under thermal radiation. Phys. Scr..

[5] Afridi M.I., Qasim M. (2018). Entropy generation in three dimensional flow of dissipative fluid. Int. J. Appl. Comput. Math..

[6] Vasu B., Ram R.C., Murthy P., Gorla R.S.R. (2017). Entropy generation analysis in nonlinear convection flow of thermally stratified fluid in saturated porous medium with convective boundary condition. J. Heat Transf..

[7] Afridi M.I., Qasim M., Shafie S. (2017). Entropy generation in hydromagnetic boundary flow under the effects of frictional and Joule heating: Exact solutions. Eur. Phys. J. Plus.

[8] Butt A.S., Ali A., Mehmood A. (2016). Entropy generation effects in Cu water nano fluid flow and heat transfer over a radially stretching surface. J. Nanofluids.

[9] Makinde O.D., Eegunjobi A.S. (2013). Entropy generation in a couple stress fluid flow through a vertical channel filled with saturated porous media. Entropy.

[10] Rashidi M.M., Abelman S., Mehr N.F. (2013). Entropy generation in steady MHD flow due to a rotating porous disk in a nanofluid. Int. J. Heat Mass Transf..

[11] Schlichting H., Gersten K. (2016). Boundary-Layer Theory.

[12] Sakiadis B.C. (1961). Boundary-layer behavior on continuous solid surfaces: I. Boundary-layer equations for two-dimensional and axisymmetric flow. AIChE J..

[13] Crane L.J. (1970). Flow past a stretching plate. J. Appl. Math. Phys..

[14] Gupta P.S., Gupta A.S. (1977). Heat and mass transfer on a stretching sheet with suction or blowing. Can. J. Chem. Eng..

[15] Wang C.Y. (1988). Fluid flow due to a stretching cylinder. Phys. Fluids.

[16] Wang C.Y. (1988). Stretching a surface in a rotating fluid. J. Appl. Math. Phys..

[17] Dandapat B.S., Santra B., Vajravelu K. (2007). The effects of variable fluid properties and thermocapillarity on the flow of a thin film on an unsteady stretching sheet. Int. J. Heat Mass Transf..

[18] Vajravelu K., Rollins D. (2004). Hydromagnetic flow of a second grade fluid over a stretching sheet. Appl. Math. Comput..

[19] Pal D., Mondal H. (2014). Effects of temperature-dependent viscosity and variable thermal conductivity on MHD non-Darcy mixed convective diffusion of species over a stretching sheet. J. Egypt. Math. Soc..

[20] Yazdi M.H., Hashim I., Sopian K. (2014). Bin Slip boundary layer flow of a power-law fluid over moving permeable surface with viscous dissipation and prescribed surface temperature. Int. Rev. Mech. Eng..

[21] Hsiao K.-L. (2008). MHD mixed convection of viscoelastic fluid over a stretching sheet with ohmic dissipation. J. Mech..

[22] Vajravelu K., Prasad K.V., Vaidya H. (2016). Influence of hall current on MHD flow and heat transfer over a slender stretching sheet in the presence of variable fluid properties. Commun. Numer. Anal..

[23] Vajravelu K., Prasad K.V., Vaidya H., Basha N.Z., Ng C.-O. (2017). Mixed convective flow of a Casson fluid over a vertical stretching sheet. Int. J. Appl. Comput. Math..

[24] Ali F., Sheikh N.A., Khan I., Saqib M. (2018). Influence of a porous medium on the hydromagnetic free convection flow of micropolar fluid with radiative heat flux. J. Porous Media.

[25] Hsiao K.-L. (2007). Conjugate heat transfer of magnetic mixed convection with radiative and viscous dissipation effects for second-grade viscoelastic fluid past a stretching sheet. Appl. Therm. Eng..

[26] Pop I., Isa S.S.P.M., Arifin N.M., Nazar R., Bachok N., Ali F.M. (2016). Unsteady viscous MHD flow over a permeable curved stretching/shrinking sheet. Int. J. Numer. Methods Heat Fluid Flow.

[27] Reddy J.V.R., Sugunamma V., Sandeep N. (2018). Dual solutions for nanofluid flow past a curved surface with nonlinear radiation, Soret and Dufour effects. J. Phys. Conf. Ser..

[28] Rudraswamy N.G., Kumar K.G., Gireesha B.J., Gorla R.S.R. (2016). Soret and Dufour effects in three-dimensional flow of Jeffery nanofluid in the presence of nonlinear thermal radiation. J. Nanoeng. Nanomanuf..

[29] Roşca N.C., Pop I. (2015). Unsteady boundary layer flow over a permeable curved stretching/shrinking surface. Eur. J. Mech..

[30] Rashad A.M., Rashidi M.M., Lorenzini G., Ahmed S.E., Aly A.M. (2017). Magnetic field and internal heat generation effects on the free convection in a rectangular cavity filled with a porous medium saturated with Cu–water nanofluid. Int. J. Heat Mass Transf..

[31] Sheikholeslami M., Vajravelu K., Rashidi M.M. (2016). Forced convection heat transfer in a semi annulus under the influence of a variable magnetic field. Int. J. Heat Mass Transf..

[32] Sheikholeslami M., Rashidi M.M., Ganji D.D. (2015). Effect of non-uniform magnetic field on forced convection heat transfer of Fe_3_O_4_–water nanofluid. Comput. Methods Appl. Mech. Eng..

[33] Shu C. (2012). Differential Quadrature and Its Application in Engineering.

[34] Baskaya E., Komurgoz G., Ozkol I. (2017). Investigation of oriented magnetic field effects on entropy generation in an inclined channel filled with ferrofluids. Entropy.

